# Hearing recovery prediction and prognostic factors of idiopathic sudden sensorineural hearing loss: a retrospective analysis with a deep neural network model

**DOI:** 10.1016/j.bjorl.2023.04.001

**Published:** 2023-04-21

**Authors:** Tae Woong Uhm, Seongbaek Yi, Sung Won Choi, Se Joon Oh, Soo Keun Kong, Il Woo Lee, Hyun Min Lee

**Affiliations:** a Department of Statistics, Pukyong National University, Busan, Republic of Korea; b Department of Otorhinolaryngology-Head and Neck Surgery, Pusan National University College of Medicine, Pusan National University Hospital, Busan, Republic of Korea; c Department of Otorhinolaryngology-Head and Neck Surgery, Pusan National University College of Medicine, Pusan National University Yangsan Hospital, Yangsan, Gyeongnam, Republic of Korea

**Keywords:** Hearing loss, sudden, Prognosis, Outcome prediction, Deep neural network

## Abstract

**Objective:**

Idiopathic Sudden Sensorineural Hearing Loss (ISSHL) is an otologic emergency, and an early prediction of prognosis may facilitate proper treatment. Therefore, we investigated the prognostic factors for predicting the recovery in patients with ISSHL treated with combined treatment method using machine learning models.

**Methods:**

We retrospectively reviewed the medical records of 298 patients with ISSHL at a tertiary medical institution between January 2015 and September 2020. Fifty-two variables were analyzed to predict hearing recovery. Recovery was defined using Siegel’s criteria, and the patients were categorized into recovery and non-recovery groups. Recovery was predicted by various machine learning models. In addition, the prognostic factors were analyzed using the difference in the loss function.

**Results:**

There were significant differences in variables including age, hypertension, previous hearing loss, ear fullness, duration of hospital admission, initial hearing level of the affected and unaffected ears, and post-treatment hearing level between recovery and non-recovery groups. The deep neural network model showed the highest predictive performance (accuracy, 88.81%; area under the receiver operating characteristic curve, 0.9448). In addition, initial hearing level of affected and non-affected ear, post-treatment (2-weeks) hearing level of affected ear were significant factors for predicting the prognosis.

**Conclusion:**

The deep neural network model showed the highest predictive performance for recovery in patients with ISSHL. Some factors with prognostic value were identified. Further studies using a larger patient population are warranted.

**Level of evidence:**

Level 4.

## Highlights

• Various machine learning methods used to predict hearing recovery in ISSHL patients.
• The deep neural network method showed the highest predictive performance.
• Initial and early post-treatment hearing levels were significant prognostic factors.
• Machine learning may help predict ISSHL prognosis, as shown in the study.

## Introduction

Idiopathic Sudden Sensorineural Hearing Loss (ISSHL) is an otological emergency that occurs within a 72 -h window and is characterized by unilateral or bilateral hearing loss of ≥30 decibels at three consecutive audiometric frequencies. Although the cause of ISSHL has not been precisely identified, it is thought to be related to viral infection, vascular impairment, or autoimmune diseases.^1^ ISSHL is generally treated with high-dose systemic steroids. However, in patients unable to use systemic steroids, Intratympanic Steroid Injection (ITSI) and hyperbaric oxygen treatment are effective as salvage treatments.^2^ When the patient does not receive proper treatments after the onset of ISSHL, the resulting hearing loss leads to decreased ability of sound localization and speech perception in noise and tinnitus, thereby deteriorating the patient’s quality of life in the long term. Hence, proper and timely treatment is very important.^3^ Notably, the prediction of prognosis may lead to better treatment results because more additional treatments, such as salvage ITSI or hyperbaric oxygen treatment, can be added when a poor prognosis is predicted.^4^ Due to developments in artificial intelligence technology, studies have been conducted to predict the prognosis of various diseases using machine learning models, including ISSHL.^5^, ^6^, ^7^, ^8^

The purpose of this study was to overcome the limitations of previous studies, analyze larger number of patients with ISSHL, and predict hearing recovery using the Deep Neural Network (DNN) model. In addition, we identified factors that had prognostic value.

## Methods

### Patient epidemiology and medical data collection

**Figure 1:**
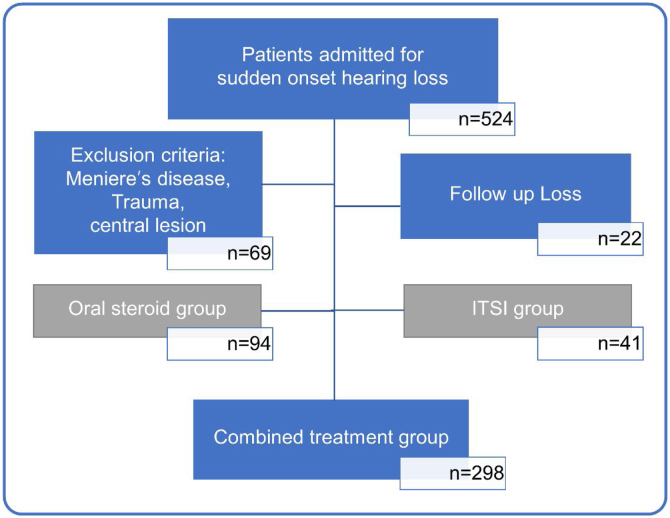
Flowchart showing the process of selecting study participants. Based on the type of initial treatment received, patients were divided into those treated with only oral steroids (oral steroid group), only initial Intratympanic Steroid Injection (ITSI) (ITSI group), and oral steroids with ITSI or additional salvage ITSI (combined treatment group). To reduce bias due to differences in treatment methods, only the combined treatment group was analyzed.

In this study, we retrospectively reviewed the medical records of 524 unilateral ISSHL patients who had received inpatient treatment at a tertiary medical center, between January 2015 and September 2020. Sixty-nine patients with sudden hearing loss due to other conditions, such as Meniere’s disease, head trauma, meningitis, and central lesions, and twenty-two patients who could not be followed up after treatment were excluded. Among 443 patients with ISSHL, 94 patients in the oral steroid group and 41 patients in the ITSI group were excluded from the analysis to reduce bias due to differences in treatment methods. Finally, 298 patients with ISSHL treated with the combined treatment method (oral steroid + ITSI) were included in the analysis (Fig. 1).

Patients’ age, height, weight, sex, Body Mass Index (BMI), history of smoking and alcohol consumption, history of otological diseases (hearing loss, chronic otitis media, tinnitus, and dizziness), current underlying diseases (hypertension, diabetes, cardiac disease, and cerebrovascular accident), initial otological symptoms accompanying hearing loss (tinnitus, ear fullness, and dizziness), duration of hospital admission, delay from symptom onset to treatment, laterality, and pure tone audiometry results (initial and post-treatment at 2-weeks and 3-months) were extracted from medical records and investigated. This study was approved by the institutional review board of the Pusan National University Yangsan Hospital (PNUYH IRB, number 15-2021-069). Obtaining informed consent was waived due to the retrospective study under the permission of the institutional review board.

### Treatment methods and hearing assessment

The patients with ISSHL in the combined treatment group were treated with oral steroids and ITSI simultaneously or with additional salvage ITSI after the oral steroid treatment. The choice of treatment was based on the initial hearing level, early hearing level after treatment, comorbidities, and preference of the patient.

In all patients, Gingko biloba (Tanamin [35 mg/10 mL], Yuyu Pharma Inc., Seoul, Korea) and carbogen inhalation therapy (95% O_2_ + 5% CO_2_, twice daily) were used during inpatient treatment, and lipoprostaglandin (Eglandin [5 μg/mL], Mitsubishi Tanabe Pharma Korea Co., Ltd, Seoul, Korea) was administered for 5 days. The oral methylprednisolone (Methylon [48 mg], Alvogenkorea, Seoul, Korea) was administered for 10 days and was then tapered to half the dosage over the next 4 days. ITSI was administered either daily or once every 2 days. The status of the patient’s tympanic membrane was checked prior to injection; the injection was not administered if the patient had otitis media, adhesions, or perforation of the tympanic membrane. The ear canal and tympanic membrane were anesthetized using an ointment (EMLA cream [5 g], AstraZeneca Korea, Seoul, Korea) for 10 min, and 0.5–0.7 cc of dexamethasone (5 mg/mL, Daewon Pharmaceutical, Seoul, Korea) was injected into the anteroinferior quadrant of the tympanic membrane using a 1-cc syringe connected to a 26-gauge spinal needle.

Siegel’s criteria were used to define hearing recovery.^9^ The average hearing threshold was calculated using the values of 500, 1000, 2000 and 3000 Hz. To confirm the relationship between the low and high frequencies during hearing recovery, the low-tone average was defined as the average of the values at 250 Hz and 500 Hz, and the high-tone average was defined as the average of the values at 4000 Hz and 8000 Hz. The degree of hearing improvement according to Siegel’s criteria was defined as follows: (I) Complete Recovery (CR), a final hearing level better than 25 dB regardless of the size of the gain; (II) Partial Recovery (PR), >15 dB of gain and a final hearing level of 25–45 dB; (III) Slight Improvement (SI), >15 dB of gain and a final hearing level worse than 45 dB; and (IV) No Improvement (NI), <15 dB of gain and a final hearing level worse than 75 dB. The difference between the initial and post-treatment (3-months) hearing thresholds was calculated based on the treatment results. Patients with CR and PR were defined as the recovery group, as described previously.^6^

### Statistical analysis and machine learning model development

The *t*-test and Chi-Square test were used to compare the homogeneity between the recovery and non-recovery groups. The level of significance was set at 0.05. Five indices were used to evaluate and compare the performance of each machine learning model. A model with index values close to 1 was considered good. The following machine learning methods were used in this study: Least Absolute Shrinkage and Selection Operator (LASSO), decision tree, support vector machine, random forest, boosting, and DNN. A DNN is an artificial neural network composed of multiple hidden layers between the input and output layers. Neurons in the input or previous hidden layers are combined with the weights of the next or previous hidden layers, and the weights of the output and previous hidden layers are adjusted according to their contribution to the loss function. The machine learning methods are described in the Appendix Asupporting information file, and the information on the number of layers and nodes in the DNN, and the hyperparameter tuning values are listed in S1 Table. Variable importance, a loss function gap between a full model and a model excluding a certain variable, was used for selecting the prognostic factors. The greater the difference in loss between the two models, the higher the variable importance. Statistical analyses were conducted using R 4.0.5 (R Foundation, Vienna, Austria) and Python 3.7 software.

## Results

The average age of all included patients was 51.7 ± 14.3 years, and the proportions of males and females were almost the same. Continuous variables, such as age, weight, height, BMI, duration of hospital admission, treatment delay from symptom onset, and hearing thresholds before and after treatment, are summarized in Table 1. Categorical variables, such as sex, history of smoking and alcohol consumption, history of otological diseases, current underlying diseases, initial otological symptoms accompanying hearing loss, and laterality, are summarized in Table 2.

**Table 1:** Clinical characteristics and hearing levels in patients with idiopathic sudden sensorineural hearing loss grouped by recovery status.

Parameter	Recovery (n = 117)	Non-recovery (n = 181)	Total (n = 298)	*p*-value
Age	48.7 ± 14.6	53.6 ± 13.8	51.7 ± 14.3	0.003
Height (cm)	164.0 ± 9.2	163.6 ± 8.8	163.7 ± 9.0	0.686
Weight (kg)	65.9 ± 13.2	66.9 ± 13.3	66.5 ± 13.2	0.517
BMI (kg/m^2^)	24.4 ± 3.8	24.8 ± 3.6	24.7 ± 3.7	0.295
Duration of hospital admission (days)	7.6 ± 2.3	8.7 ± 2.8	8.3 ± 2.6	<0.001
Delay from symptom onset to treatment (days)	5.2 ± 7.8	6.8 ± 10.1	6.2 ± 9.3	0.145
Initial average hearing threshold of AE (dB)	63.3 ± 21.9	84.9 ± 28.8	76.4 ± 28.3	<0.001
Initial hearing threshold of AE by frequency (dB)				
250 Hz	57.1 ± 26.8	77.8 ± 31.4	69.7 ± 31.3	<0.001
500 Hz	65.4 ± 24.2	82.3 ± 29.6	75.7 ± 28.8	<0.001
1000 Hz	67.2 ± 24.8	86.3 ± 29.0	78.8 ± 28.9	<0.001
2000 Hz	60.9 ± 25.7	84.9 ± 31.8	75.5 ± 31.8	<0.001
3000 Hz	59.6 ± 23.5	86.2 ± 31.3	75.7 ± 31.3	<0.001
4000 Hz	63.2 ± 24.6	89.1 ± 30.0	78.9 ± 30.7	<0.001
8000 Hz	65.4 ± 27.6	95.4 ± 39.4	83.6 ± 38.1	<0.001
Low-tone average	61.2 ± 24.3	80.0 ± 29.8	72.7 ± 29.2	<0.001
High-tone average	64.3 ± 25.0	92.2 ± 32.2	81.2 ± 32.5	<0.001
Initial average hearing threshold of NAE (dB)	16.1 ± 18.0	25.4 ± 26.1	21.8 ± 23.7	<0.001
Initial hearing threshold of NAE by frequency (dB)				
250 Hz	14.0 ± 17.0	23.2 ± 24.9	19.6 ± 22.6	<0.001
500 Hz	14.0 ± 17.5	22.1 ± 25.5	18.9 ± 23.0	0.001
1000 Hz	15.3 ± 18.2	23.7 ± 26.5	20.4 ± 23.9	0.001
2000 Hz	16.7 ± 18.5	24.8 ± 27.3	21.6 ± 24.5	0.003
3000 Hz	18.4 ± 21.3	30.3 ± 29.4	25.7 ± 27.1	<0.001
4000 Hz	24.0 ± 23.6	35.6 ± 30.2	31.0 ± 28.3	<0.001
8000 Hz	30.1 ± 26.8	42.0 ± 32.4	37.4 ± 30.8	0.001
Low-tone average	14.0 ± 16.9	22.6 ± 24.9	19.2 ± 22.5	<0.001
High-tone average	27.1 ± 24.1	38.8 ± 30.4	34.2 ± 28.6	<0.001
Post-treatment (2-weeks) average hearing threshold of AE (dB)	26.1 ± 14.4	75.0 ± 28.0	55.8 ± 33.6	<0.001
Post-treatment (2-weeks) hearing threshold of AE by frequency (dB)				
250 Hz	20.6 ± 14.7	64.8 ± 33.3	47.5 ± 35.0	<0.001
500 Hz	23.7 ± 15.9	70.6 ± 30.0	52.2 ± 34.2	<0.001
1000 Hz	26.5 ± 16.7	75.6 ± 29.7	56.4 ± 34.9	<0.001
2000 Hz	24.6 ± 18.6	75.2 ± 30.8	55.3 ± 36.4	<0.001
3000 Hz	29.7 ± 22.1	78.8 ± 30.1	59.5 ± 36.3	<0.001
4000 Hz	37.4 ± 24.8	82.6 ± 29.0	64.9 ± 35.2	<0.001
8000 Hz	46.1 ± 28.8	89.3 ± 28.9	72.3 ± 35.7	<0.001
Low-tone average	22.2 ± 14.3	67.7 ± 31.0	49.8 ± 34.0	<0.001
High-tone average	41.8 ± 25.5	86.0 ± 28.2	68.6 ± 34.7	<0.001
Post-treatment (3-month) average hearing threshold of AE (dB)	21.4 ± 10.8	69.1 ± 23.1	50.4 ± 30.2	< 0.001
Post-treatment (3-month) low-tone average hearing threshold of AE (dB)	17.4 ± 10.3	58.2 ± 28.7	42.2 ± 30.7	<0.001
Post-treatment (3-month) high-tone average hearing threshold of affected ear (dB)	37.9 ± 24.3	81.0 ± 25.8	64.1 ± 32.8	<0.001

Values are presented as mean ± standard deviation. The low tone average means the hearing average at 250 Hz and 500 Hz, and the high tone average means the hearing average at 4 kHz and 8 kHz.

BMI, Body Mass Index; AE, Affected Ear; NAE, Non-Affected Ear.

**Table 2:** Clinical categorical variables and hearing recovery in patients with idiopathic sudden sensorineural hearing loss.

Variables		Recovery (n = 117)	Non-recover (n = 181)	Total (n = 298) (%)	Recovery rate (%)	*p-*value
Sex	Male	56	94	150 (50.3)	37.3	0.570
	Female	61	87	148 (49.7)	41.2	
Alcohol consumption	(-)	73	125	198 (66.4)	36.9	0.287
	(+)	44	56	100 (33.6)	44.0	
Smoking	(-)	95	153	248 (83.2)	38.3	0.553
	(+)	22	28	50 (16.8)	44.0	
History of hearing loss	(-)	100	116	216 (72.5)	46.3	<0.001
	(+)	17	65	82 (27.5)	20.7	
History of chronic otitis media	(-)	113	168	281 (94.3)	40.2	0.266
	(+)	4	13	17 (5.7)	23.5	
History of tinnitus	(-)	110	165	275 (92.3)	40.0	0.496
	(+)	7	16	23 (7.7)	30.4	
History of dizziness	(-)	112	171	283 (95.0)	39.6	0.833
	(+)	5	10	15 (5.0)	33.3	
Hypertension	(-)	95	126	221 (74.2)	43.0	0.036
	(+)	22	55	77 (25.8)	28.6	
Diabetes	(-)	105	161	266 (89.3)	39.5	0.981
	(+)	12	20	32 (10.7)	37.5	
Cardiac disease	(-)	116	173	289 (97.0)	40.1	0.159
	(+)	1	8	9 (3.0)	11.1	
CVA	(-)	117	179	296 (99.3)	39.5	0.679
	(+)	0	2	2 (0.7)	0.0	
Tinnitus	(-)	24	47	71 (23.8)	33.8	0.347
	(+)	93	134	227 (76.2)	41.0	
Ear fullness	(-)	46	103	149 (50.0)	30.9	0.004
	(+)	71	78	149 (50.0)	47.7	
Dizziness	(-)	94	97	191 (64.1)	49.2	<0.001
	(+)	23	84	107 (35.9)	21.5	
Laterality	Right	46	83	129 (43.3)	42.0	0.321
	Left	71	98	169 (56.7)	35.7	

CVA, Cerebrovascular Accident.

In all patients, the initial average hearing thresholds of the affected and unaffected ears were 76.4 ± 28.3 dB and 21.8 ± 23.7 dB, respectively. The average hearing thresholds of the affected ear at 2-weeks and 3-months post-treatment were 55.8 ± 36.6 dB and 50.4 ± 30.2 dB, respectively. The initial and post-treatment hearing thresholds of the affected ear and the initial hearing threshold of the non-affected ear were significantly better in the recovery group than in the non-recovery group (Table 1).

Significant differences in specific variables were evaluated between the two groups. Patients in the recovery group were significantly younger than those in the non-recovery group. The duration of hospital admission was significantly longer in the non-recovery group than in the recovery group. There were significantly more patients with a history of hearing loss, hypertension, and dizziness as an accompanying symptom in the non-recovery group. In contrast, the number of patients with ear fullness as an accompanying symptom was significantly higher in the recovery group.

**Figure 2:**
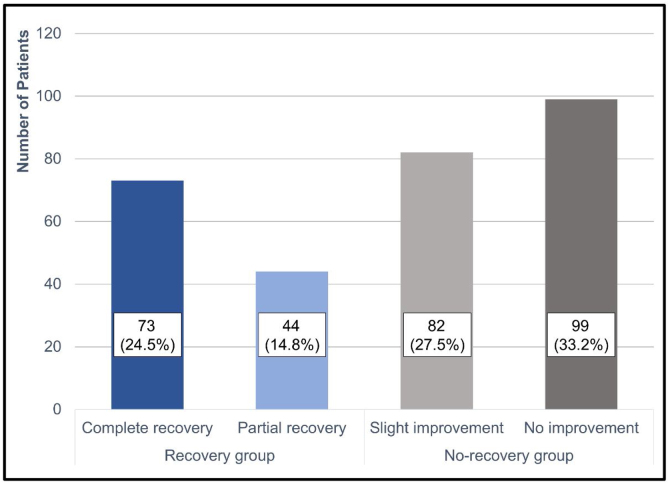
Hearing recovery in idiopathic sudden sensorineural hearing loss patients according to Siegel’s criteria.

**Figure 3:**
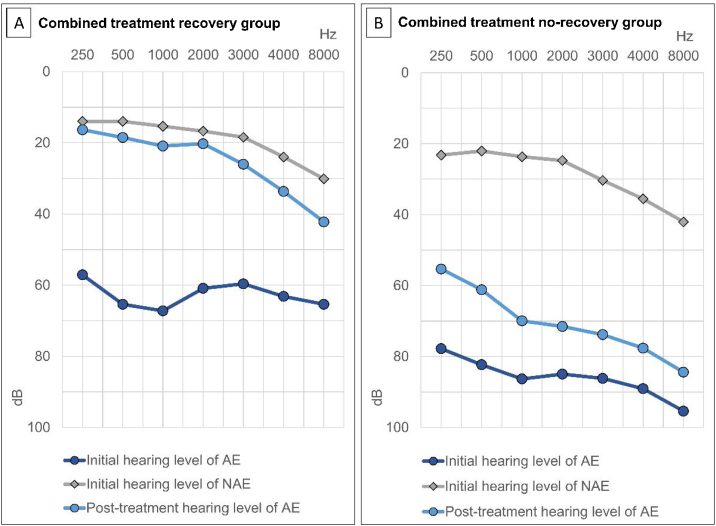
Initial and post-treatment average hearing levels in the affected and non-affected ears according to the recovery status. (A) Combined treatment recovery group. (B) Combined treatment non-recovery group. AE, Affected Ear; NAE, Non-Affected Ear.

Treatment outcomes based on Siegel’s criteria were as follows: CR, PR, SI, and NI were achieved in 73 (24.5%), 44 (14.8%), 82 (27.5%), and 99 (33.2%) patients, respectively (Fig. 2). The recovery rate by defining CR and PR as recovery was 39.3%. According to the two groups, the average hearing threshold at each frequency was calculated (Fig. 3).

The performance of various machine learning methods in predicting the prognosis of ISSHL is summarized in Table 3. The DNN model showed the highest predictive power [accuracy, 88.81%; area under the receiver operating characteristic curve (AUC), 0.9448], followed by the random forest model (accuracy, 86.76%; AUC, 0.9442).

**Table 3:** Hearing recovery prediction performance of LASSO, decision tree, SVM, random forest, boosting, and DNN methods.

	Accuracy (%)	F-score	ROC-AUC	95% CI for the ROC-AUC	Precision (%)	Recall (%)
LASSO	84.88	0.8060	0.9271	0.9239‒0.9303	81.10	80.60
Decision tree	83.72	0.7855	0.8657	0.8609‒0.8704	80.87	77.82
SVM	83.47	0.7780	0.8093	0.7323‒0.8864	82.28	74.76
Random forest	86.76	0.8328	0.9442	0.9423‒0.9462	82.46	84.59
Boosting	85.90	0.8203	0.9287	0.9259‒0.9315	81.93	82.75
DNN	88.81	0.8868	0.9448	0.9359‒0.9521	87.95	88.67

ROC-AUC, Area Under the Receiver Operating Characteristic Curve; CI, Confidence Interval; LASSO, Least Absolute Shrinkage and Selection Operator; SVM, Support Vector Machine; DNN, Deep Neural Network.

To identify the significant prognostic factors for recovery from ISSHL, the variable importance, which is the difference in loss function between models based on the presence or absence of a specific variable, was calculated in the DNN method. If the value of the variable importance becomes negative, it can be judged as a significant factor for predicting the prognosis. According to the analysis, initial hearing level of the affected ear and non-affected ear, post-treatment (2-weeks) hearing level of the affected ear, smoking, tinnitus, laterality, and BMI were significant factors for predicting the prognosis of ISSHL (Table 4, S2 Table). The whole variable importance values are indicated in S2 Table.

**Table 4:** Top 10 significant variables from the deep neural network.

Variables	Loss	Variable importance
DNN full model	0.6891	
Initial hearing threshold of AE at 3 kHz	1.0561	−0.3670
Initial hearing threshold of NAE at 250 Hz	0.9157	−0.2266
Initial hearing threshold of AE at 4 kHz	0.8926	−0.2035
Initial hearing threshold of AE at 2 kHz	0.8784	−0.1893
Post-treatment (2-weeks) hearing threshold of AE at 500 Hz	0.8708	−0.1817
Post-treatment (2-weeks) hearing threshold of AE at 3 kHz	0.8609	−0.1717
Post-treatment (2-weeks) hearing threshold of AE at 2 kHz	0.8361	−0.1470
Post-treatment (2-weeks) average hearing threshold of AE	0.7838	−0.0947
Post-treatment (2-weeks) hearing threshold of AE at 4 kHz	0.7779	−0.0887
Initial low tone hearing average of AE	0.7586	−0.0695

For the other variable values, the differences of loss were positive numbers and were not important for prediction; therefore, they have been separately added to the supplement information file.

DNN, Deep Neural Network; AE, Affected Ear; NAE, Non-Affected Ear.

## Discussion

The prognosis of ISSHL is generally predicted using conventional statistical models, such as logistic regression. Recently, studies have utilized machine learning methods to better predict the prognosis of ISSHL.^5^, ^6^, ^7^, ^8^ In this study, we attempted to predict the prognosis of patients with ISSHL using various machine learning methods based on clinical characteristics. Significant differences in various factors were identified between the recovery and non-recovery groups. As demonstrated previously,^10^, ^11^, ^12^, ^13^ patients in the recovery group were younger than those in the non-recovery group.

In our study, hypertension was more common in the non-recovery group. On the other hand, hypertension was not a significant prognostic factor in the DNN method. Some previous studies showed poor prognosis in patients with hypertension,^10^, ^12^ although other studies found that the presence of hypertension was not a prognostic factor.^13^, ^14^ In hypertensive patients, the blood vessel elasticity of the inner ear may decrease, thereby causing atherosclerotic change, which may narrow the blood vessels and aggravate damage to the inner ear.^15^ In addition, we found no differences in the presence of diabetes between the recovery and non-recovery groups. Although some studies showed similar results,^12^, ^14^ other studies demonstrated poor prognosis in the presence of hyperglycemia.^13^, ^16^ Regarding the relationship between other systemic diseases and the recovery from ISSHL, poor recovery of hearing loss has been reported in the presence of the metabolic syndrome.^15^ Some studies have suggested that cardiac disease or cardiovascular accident is not a prognostic factor for ISSHL;^12^, ^13^ however, others have reported an increased risk of stroke and myocardial infarction after the onset of ISSHL.^17^ It has also been hypothesized that cochlear microangiopathy due to endothelial vascular abnormalities may affect the prognosis of ISSHL in the presence of comorbidities, such as diabetes or metabolic syndrome.^15^ Thus, there is controversy on whether underlying diseases such as hypertension and diabetes affect the prognosis in patients with ISSHL. Since the characteristics of the target group vary between studies, the association between the underlying disease and ISSHL remains to be further investigated.

Regarding the early accompanying otologic symptoms associated with ISSHL, we found that there were more patients with ear fullness in the recovery group, and no difference in the number of patients with tinnitus was observed between the recovery and non-recovery groups. Tinnitus was identified as a possible prognostic factor for predicting the prognosis of ISSHL in the DNN method. Previous studies showed that the prognosis was usually better when tinnitus and ear fullness were present,^12^, ^18^ probably because these symptoms can be recognized more quickly and treatment can be initiated early. Tinnitus indicates the presence of residual hearing,^19^ and ISSHL patients with dizziness as an initial accompanying symptom often show a poor prognosis.^11^, ^13^, ^14^, ^20^^,^^21^ Notably, it has been suggested that the cochlea and vestibule receive blood supply from the internal auditory artery; therefore, when ischemia occurs, both parts are affected, resulting in a poor prognosis in ISSHL.^21^ Likewise, we found that recovery was poor when dizziness was an initial accompanying symptom. However, dizziness was not a significant prognostic factor of ISSHL in the DNN method. Therefore, additional research is needed to confirm its role as a prognostic factor.

A poor initial hearing threshold of the affected ear has been demonstrated to be associated with a poor prognosis.^11^, ^13^, ^20^ In this study, both initial and post-treatment (2-weeks) hearing thresholds of the affected ear were better in the recovery group. Initial hearing threshold and post-treatment (2-weeks) hearing thresholds of the affected ear were also identified as significant prognostic factors of ISSHL in the DNN method. A similar trend was observed in previous studies: higher hearing recovery rates were observed in groups showing quick hearing recovery within 7-days,^11^, ^22^ or 2 weeks.^23^ Moreover, we found that there were more patients with a previous history of hearing loss, and the initial hearing threshold of the non-affected ear was worse in the non-recovery group than that in the recovery group. Furthermore, the initial hearing threshold of the non-affected ear was a significant prognostic factor based on the DNN model. This could be due to an existing underlying bilateral hearing dysfunction or systemic disorder.^20^

Some variables require additional considerations in this study. First, studies have demonstrated that a shorter delay between symptom onset and treatment leads to a better prognosis.^13^, ^16^, ^20^ In this study, we could not find any differences between the recovery group and the non-recovery group in the delay between symptom onset and treatment. Second, the duration of hospital admission was significantly longer in the non-recovery group than in the recovery group, probably because additional ITSI is often performed when hearing recovery is not observed during hospitalization. Third, in general, additional treatments documented in this study are not well-accepted currently, although some studies have implied that additional treatments, such as lipo-prostaglandins, may affect the prognosis.^14^

In this study, we found that the DNN method had a better predictive performance than the approaches used in a similar previous study.^7^ There are several reasons for its better predictive performance. First, the sample size in this study was relatively larger than that in the former study; a larger sample size enhances the prediction performance.^24^, ^25^ Second, more independent variables were analyzed. The DNN method has better predictive performance than conventional statistical methods and has been widely used recently for prediction and classification in the medical research field.^16^, ^26^ According to the previous studies,^5^, ^6^, ^8^, ^27^^,^^28^ among machine learning methods, DNN is known to have the best predictive power. However, this method is a so-called “black box” artificial intelligence model, and although its predictive performance is high, it is not clearly known how it works or why the performance is high.^29^ Therefore, in this study, we used the loss function based on a previous study^27^ to identify significant prognostic factors and pursue an explainable artificial intelligence model. Variables with negative variable importance (initial hearing level of affected and non-affected ear, post-treatment [2-weeks] hearing level of affected ear, smoking, tinnitus, laterality, and BMI) were considered significant prognostic factors.

This study has several limitations. First, although the sample size was larger than those in the previous study, it was still small for predicting the prognosis with accuracy using a machine learning algorithm. In this study, to increase the number of patients included in the analysis, the ITSI group or oral steroid group was included initially in the study. However, since the clinical characteristics of the patient group according to the treatment method were different, it was judged that it would bias the prognosis predictions; therefore, only the combined treatment group was analyzed. The higher the number of patients, the more precise the prediction; therefore, analysis using well-organized datasets containing more patients selected through well-planned multicenter studies are needed. Second, we only evaluated simple categorical variables, general demographics, and pre- and post-treatment hearing threshold levels. To overcome these limitations, it is necessary to utilize more diverse variables. Recent studies have evaluated auditory brainstem response, otoacoustic emissions,^30^ neutrophil-to-lymphocyte ratio, and C-reactive protein-albumin ratio^31^, ^32^ in patients with ISSHL and vestibular function in patients with ISSHL with dizziness^21^ to predict the prognosis. Therefore, analyzing these variables in future studies may allow the prediction of disease prognosis with better accuracy. Third, in predicting the recovery of ISSHL, the definition of recovery and non-recovery was analyzed as a categorical variable. A previous study has reported a method to calculate the numerical recovery rate by comparing the pre- and post-treatment hearing levels of the affected ear and the contralateral hearing level.^20^ However, in this study, the difference in loss function was used to identify the importance of factors for predicting prognosis in the DNN method. The application of the numerical recovery rate required an overly complicated process to identify significant factors for predicting prognosis in the DNN method; therefore, the method described in this study was used instead. Lastly, since Gingko Biloba, lipo-prostaglandin, and carbogen are not generally accepted treatments, the treatment outcomes observed in this study should be interpreted with caution.

## Conclusion

In conclusion, we evaluated multiple variables to predict the treatment prognosis using machine learning methods in patients with ISSHL. The DNN method showed the highest predictive power (accuracy: 88.81%, AUC = 0.9448) and was considered the most useful for prognostic prediction. Initial hearing level of affected and non-affected ear, post-treatment (2-weeks) hearing level of affected ear were significant prognostic factors. Our prognosis prediction model could help design an appropriate treatment plan for patients with ISSHL. Further studies with more patients, more analyzed variables, and randomly assigned treatment methods should be undertaken.

## Authors' contributions

Conceptualization: UTW and HML. Data curation: TWU and SBY. Formal analysis: TWU, SWC and HML. Funding acquisition: HML.dd Methodology: TWU, SJO and HML. Project administration: HML and IWL. Advising: SKK and IWL. Visualization: TWU and HML. Writing-original draft: TWU and HML Writing-review & editing: TWU and HML.

## Funding

This study was supported by Research institute for Convergence of biomedical science and technology (30-2017-015), Pusan National University Yangsan Hospital, Yangsan, Republic of Korea.

## Conflicts of interest

The authors declare no conflicts of interest.
