# Supplementary material for: Hearing recovery prediction and prognostic factors of idiopathic sudden sensorineural hearing loss: a retrospective analysis with a deep neural network model
Source: Braz J Otorhinolaryngol. 2023 Apr 21;89(4):101273. doi: 10.1016/j.bjorl.2023.04.001 (PMC10391245; doi:10.1016/j.bjorl.2023.04.001)
Supplement: Supplementary file 1 [file mmc1.pdf]

Supporting information

Hearing recovery prediction and prognostic factors of idiopathic sudden sensorineural hearing loss: A retrospective analysis with a deep neural network model

This supporting information describes the machine learning model methodology used in this study.

Least absolute shrinkage and selection operator(LASSO)

The LASSO is a linear regression model that uses L1 regularization; it completely removes the weights of less important variables. $\hat{\beta}_{LASSO}(\lambda)$, which is a LASSO coefficient, can be expressed as follows:

$$\hat{\beta}_{LASSO}(\lambda) = \underset{\boldsymbol{\beta}}{argmin}\ (\boldsymbol{y} - \boldsymbol{X\beta})'(\boldsymbol{y} - \boldsymbol{X\beta})\ subject\ to\ \sum_{j=1}^{p} |\beta_j| \leq s$$

$$= \underset{\boldsymbol{\beta}}{argmin}\ (\boldsymbol{y} - \boldsymbol{X\beta})'(\boldsymbol{y} - \boldsymbol{X\beta}) + \lambda \sum_{j=1}^{p} |\beta_j|$$

where the l_1 norm of the coefficient vector β means $||\boldsymbol{\beta}||_1 = \sum |\beta_j|$. λ, which is a tuning parameter of the l_1 penalty, is a value to be estimated. LASSO minimizes coefficient estimates. If λ of the tuning parameter is sufficiently large, a set of the coefficient estimates will have a value of exactly 0 and will have the effect of variable selection. Hence, LASSO benefits from being easily interpretable.

Decision Tree

The Decision tree analysis expresses decision rules as a tree structure and classifies the entire dataset according to specific criteria. The tree-based method can be applied to regression and classification problems. When analyzing binary data, a classification tree (called a decision tree) will be used. The classification tree predicts the areas that contain the most observations. The classification tree divides the tree using two methods. The first is the Gini index, which is defined with the following formula for K classes:

$$G = \sum_{k=1}^{K} \hat{p}_{nk} \left(1 - \hat{p}_{nk}\right)$$

where $\hat{p}_{nk}$ indicates the ratio of observations belonging to the k category among the observations belonging to the n-th node. The second is cross-entropy, defined as follows:

$$E = -\sum_{k=1}^{K} \hat{p}_{nk} \log_2(\hat{p}_{nk})$$

The larger the Gini index and cross-entropy, the greater the heterogeneity within the node. Hence, the division is performed in a direction in which this value becomes the smallest. In addition to being easy to interpret, the decision tree is advantageous in that qualitative predictors can be easily inferred without creating dummy variables.

Random Forest

Ensemble methods create multiple predictive models with given materials and then combine them to create a final predictive model. A random forest is an ensemble method based on a decision tree similar to bagging: the fitting of multiple models from the bootstrap material at

$b = 1, 2, \ldots, B$ obtained using the training set. After obtaining $\hat{f}^{*(b)}(x)$, a majority vote can be performed to obtain a final predicted value as follows (in the case of a classification analysis):

$$\hat{f}_{bag}(x) = \{\hat{f}^{*(b)}(x), b = 1, 2, \ldots, B\}$$

However, unlike bagging, when dividing a branch in each decision tree, m variables of the total p variables are randomly selected. These methods remove the correlation of the trees and stabilize the model. Further, because of the maximized diversity, it features very good predictive power.

Boosting

While boosting functions similar to bagging, it allows for trees to be created sequentially – i.e., the new model is adapted using the current model's residual as the response variable. Hence, many models must be fitted. Boosting changes the model improvement speed according to a certain shrinkage estimator (λ) and controls the complexity of the boosted ensemble in each tree based on the number of divisions (d; if d = 1, it usually operates normally). In addition, if the number of trees is too large in boosting, overfitting can be performed.

Support vector machine (SVM)

Support vector machine (SVM) is a method of finding a hyperplane that can classify a data class in space. SVM is used for both classification and regression and is mainly applied to binary classification. The purpose of SVM is to find a hyperplane that can distinguish two groups with the maximum margin. A vector located on the margin boundary is called a support vector. If training data can be linearly separated, the problem for finding the optimal hyperplane

can be defined as follows for n data (x_i, y_i):

$$\min_{w,b} \frac{1}{2} \|w\|^2$$

$$\text{s.t.}\, y_i(w^T x_i + b) \geq 1, \forall i = 1, \dots, n$$

where w, b, and y_i indicate weighted vectors, bias, and data attributes, respectively. $y_i(w^T x_i + b)$ is a functional margin. The optimization problem can be solved using the Lagrange multiplier method. However, when linear separability is not performed, the problem can be solved using L_1-regularization, as in the following equation:

$$\min_{\gamma w,b} \frac{1}{2} \|w\|^2 + C \sum_{i=1}^{n} \xi_i$$

$$\text{s.t.}\, y_i(w^T x_i + b) \geq 1 - \xi_i, \forall i = 1, \dots, n$$

$$\xi_i \geq 0, \forall i = 1, \dots, n$$

Since the SVM is a black box model, the learning and classification results cannot be properly described. However, it is easy to use and features high prediction accuracy.

Deep Neural Network (DNN)

The structure of a neural network comprises a hierarchical arrangement of an input layer, a hidden layer, and an output layer. When the number of hidden layers is two or more, the neural network is known as a deep neural network. As shown in Equation (1), the input value of the hidden layer is propagated to the next layer through weighted sum calculation and activation function.

$$y = f\left(\sum_{i=1}^{n} w_i x_i + b\right) \quad (1)$$

where x_i is the input value, y is the output value, w_i is the weight, and b is the bias.

Learning of a neural network is the process of updating weights to reduce an error, which is the difference between an output value and a predicted value. The error is calculated using a loss function. To optimize the error and improve the performance of the deep neural network, the error information is back-propagated to the input layer, and each weight and bias are updated. The hyperparameters used in this paper are shown in Supplementary Table 1.

S1 Table. Setting of hyperparameters in a deep neural network

Hyperparameters	Values
Number of hidden layers	3
Weight initialization	He Uniform Initialization: $U \sim \left(-\sqrt{\frac{6}{n_{in}}}, \sqrt{\frac{6}{n_{in}}}\right)$, n_{in} is the number of previous nodes
Optimization	Adam (Adaptive moment estimation)
Hidden layer activation function	ReLU (Rectified linear unit)
Number of nodes	230
Batch size	32
Learning rate	0.1%
Epoch	70
Output layer activation function	Sigmoid function

S2 Table. Variable importance from the deep neural network

Variables	Loss	Variable importance
DNN full model	0.6891	
Initial hearing threshold of AE at 3kHz	1.0561	-0.3670
Initial hearing threshold of NAE at 250Hz	0.9157	-0.2266
Initial hearing threshold of AE at 4kHz	0.8926	-0.2035
Initial hearing threshold of AE at 2kHz	0.8784	-0.1893
Post-treatment (2 weeks) hearing threshold of AE at 500Hz	0.8708	-0.1817
Post-treatment (2 weeks) hearing threshold of AE at 3kHz	0.8609	-0.1717
Post-treatment (2 weeks) hearing threshold of AE at 2kHz	0.8361	-0.1470
Post-treatment (2 weeks) average hearing threshold of AE	0.7838	-0.0947
Post-treatment (2 weeks) hearing threshold of AE at 4kHz	0.7779	-0.0887
Initial low tone hearing average of AE	0.7586	-0.0695
Initial hearing threshold of AE at 500Hz	0.7565	-0.0674
Initial hearing threshold of NAE at 500Hz	0.7414	-0.0523
Post-treatment (2 weeks) low tone hearing average of AE	0.7388	-0.0497
Initial hearing threshold of NAE at 4kHz	0.7261	-0.0370
Smoking	0.7235	-0.0344
Initial average hearing threshold of AE	0.7215	-0.0324
Initial hearing threshold of NAE at 8kHz	0.7194	-0.0303
Initial hearing threshold of AE at 250Hz	0.7182	-0.0291
Tinnitus	0.7162	-0.0271
Laterality	0.7159	-0.0267
Initial average hearing threshold of NAE	0.7080	-0.0188
Post-treatment (2 weeks) hearing threshold of AE at 8kHz	0.7068	-0.0177
Initial hearing threshold of AE at 1kHz	0.7067	-0.0176
Initial hearing threshold of NAE at 1kHz	0.7004	-0.0113
BMI	0.6912	-0.0021

Height	0.6834	0.0057
Post-treatment (2 weeks) hearing threshold of AE at 1kHz	0.6818	0.0073
Cardiac disease	0.6780	0.0111
Diabetes	0.6776	0.0115
Post-treatment (2 weeks) high tone hearing average of AE	0.6508	0.0383
Weight	0.6475	0.0416
Dizziness	0.6473	0.0418
Initial high tone hearing average of AE	0.6431	0.0460
Ear fullness	0.6421	0.0470
Age	0.6419	0.0472
CVA	0.6410	0.0481
Initial hearing threshold of NAE at 2kHz	0.6399	0.0492
Sex	0.6389	0.0502
Post-treatment (2 weeks) hearing threshold of AE at 250Hz	0.6358	0.0534
History of chronic otitis media	0.6219	0.0672
Duration of hospital admission	0.6199	0.0692
Initial high tone hearing average of NAE	0.6171	0.0720
Initial hearing threshold of AE at 8 kHz	0.6124	0.0767
Delay from symptom onset to treatment	0.6030	0.0861
Alcohol consumption	0.5907	0.0984
History of hearing loss	0.5876	0.1015
History of dizziness	0.5821	0.1070
History of tinnitus	0.5770	0.1121
Hypertension	0.5750	0.1141
Initial hearing threshold of NAE at 3kHz	0.5695	0.1196
Initial low tone hearing average of NAE	0.5329	0.1562

Abbreviations: DNN, deep neural network; AE, affected ear; NAE, non-affected ear; BMI, Body mass index; CVA, cerebrovascular accident.
